# Development of an Ontology for Periodontitis

**DOI:** 10.1186/s13326-015-0028-y

**Published:** 2015-07-01

**Authors:** Asami Suzuki, Takako Takai-Igarashi, Jun Nakaya, Hiroshi Tanaka

**Affiliations:** Department of Computational Biology, Graduate School of Biomedical Science, Tokyo Medical and Dental University, Bunkyō, Japan; General Dentistry, The Nippon Dental University Hospital at Tokyo, Tokyo, Japan; Tohoku Medical Megabank Organization, Tohoku University, Sendai, Japan; School of Medicine, Tohoku University, Sendai, Japan

**Keywords:** ontology, periodontitis, biomedical process, bone remodeling, Gene Ontology, osteoimmunology

## Abstract

**Background:**

In the clinical dentists and periodontal researchers’ community, there is an obvious demand for a systems model capable of linking the clinical presentation of periodontitis to underlying molecular knowledge. A computer-readable representation of processes on disease development will give periodontal researchers opportunities to elucidate pathways and mechanisms of periodontitis. An ontology for periodontitis can be a model for integration of large variety of factors relating to a complex disease such as chronic inflammation in different organs accompanied by bone remodeling and immune system disorders, which has recently been referred to as osteoimmunology.

**Methods:**

Terms characteristic of descriptions related to the onset and progression of periodontitis were manually extracted from 194 review articles and PubMed abstracts by experts in periodontology. We specified all the relations between the extracted terms and constructed them into an ontology for periodontitis. We also investigated matching between classes of our ontology and that of Gene Ontology Biological Process.

**Results:**

We developed an ontology for periodontitis called Periodontitis-Ontology (PeriO). The pathological progression of periodontitis is caused by complex, multi-factor interrelationships. PeriO consists of all the required concepts to represent the pathological progression and clinical treatment of periodontitis. The pathological processes were formalized with reference to Basic Formal Ontology and Relation Ontology, which accounts for participants in the processes realized by biological objects such as molecules and cells. We investigated the peculiarity of biological processes observed in pathological progression and medical treatments for the disease in comparison with Gene Ontology Biological Process (GO-BP) annotations. The results indicated that peculiarities of Perio existed in 1) granularity and context dependency of both the conceptualizations, and 2) causality intrinsic to the pathological processes. PeriO defines more specific concepts than GO-BP, and thus can be added as descendants of GO-BP leaf nodes. PeriO defines causal relationships between the process concepts, which are not shown in GO-BP. The difference can be explained by the goal of conceptualization: PeriO focuses on mechanisms of the pathogenic progress, while GO-BP focuses on cataloguing all of the biological processes observed in experiments. The goal of conceptualization in PeriO may reflect the domain knowledge where a consequence in the causal relationships is a primary interest. We believe the peculiarities can be shared among other diseases when comparing processes in disease against GO-BP.

**Conclusions:**

This is the first open biomedical ontology of periodontitis capable of providing a foundation for an ontology-based model of aspects of molecular biology and pathological processes related to periodontitis, as well as its relations with systemic diseases. PeriO is available at http://bio-omix.tmd.ac.jp/periodontitis/.

**Electronic supplementary material:**

The online version of this article (doi:10.1186/s13326-015-0028-y) contains supplementary material, which is available to authorized users.

## Background

Biological high throughput analysis generates huge amounts of biomedical data that can be used for investigating disease mechanisms, and semantic technologies are therefore expected to contribute to the effective use of these data. Many biomedical ontologies such as the Gene Ontology (GO), the Disease Ontology (DO), the Ontology for General Medical Science (OGMS), the Human Disease Ontology (DOID) and the Infectious Disease Ontology (IDO) have been developed to provide support for sophisticated biomedical information systems [1, 2]. These ontologies are providing a means for the consistent representation of scientific data and the domain entities made possible by these data [3].

Disease has been one of the major targets for ontology development. The Unified Medical Language System (UMLS) [4] and the Medical Subject Headings (MeSH) , a source vocabulary included in UMLS, [5] are long established thesauri that explicate numerous medical terms. For many decades, the World Health Organization has provided International Classification of Diseases (ICD) [6]. Terminologies and ontologies in biology and medicine have also been reviewed by Freitas *et al.* [7]. Meanwhile, large-scale genomic projects, including the SNP consortium [8], the ENCODE project [9], the NIH Knockout Mouse Project [10], the Welcome Trust Case Control Consortium [11], and the 1000 Genome Project [12], have tried to catalogue comprehensive relationships between genes and diseases. These data collections require the development of ontologies that integrate genes with clinical outcomes [13–20]. Masci *et al.* created comprehensive definitions of dendritic cells in order to distinguish many derivatives of dendritic cells according to the progression of immune responses [20]. Mungall *et al.* investigated ontological mapping of mutation phenotypes/diseases across species [14]. Rubin *et al.* integrated existing ontologies for neuronal connectivity in order to explicate abnormalities of neuronal diseases systematically [16]. Feltrin *et al.* and Lindeberg *et al.* expanded GO in order to explicate muscle biology and plant pathology by adding specifications of pathological disorders and mutational phenotypes, respectively [17–19].

GO is an established ontology that consists of the following three sub-ontologies: Cellular Component (CC), the parts of a cell or its extracellular environment; Molecular Function (MF), the elemental activities of a gene product at the molecular level; and Biological Process (BP), operations or sets of molecular events with a defined beginning and end, pertinent to the functioning of integrated living units such as cells, tissues, organs, and organisms. While GO classifies internal processes in any biological phenomenon with external links to entries in the databases of genes by relationships of *‘associated-to’*, a specific relationship to GO [21,22], disease-centered ontologies (DO, OGMS, DOID, and IDO) only describe relationships between the processes and external perturbations, including pathogens, drugs, environmental factors, and medical devices, on the diseases. As many bio-medical researchers strive to understand diseases in the context of networks and pathways in order to realize better and personalized diagnoses and treatments in clinical medicine, molecular interpretations of both the external causes and the internal processes of disease have demanded biological high throughput analyses in order to elucidate molecular mechanisms of pathogenesis and progression. GO is currently used in biological high throughput analyses by detecting overrepresented GO terms in case groups relative to a control group [23–25]; however, we do not consider that GO covers all the required internal processes of disease, because internal disease processes may be different from the processes observed under healthy conditions. The difference can be observed not only in the classification of the internal processes, but also in the relationships between the internal processes and their participants and activators and/or suppressors to these processes [26]. Some ontologies have attempted to include annotations of genes associated with internal processes specific to the diseases such as an ontology for diabetes [2, 27]; however, those ontologies cover only a small number of existent diseases.

Periodontitis is a multifactorial disease causing inflammation in periodontal tissue. The pathogenesis of periodontitis includes numerous biological entities such as oral microorganisms and immune and genetic factors, physical effects such as dental occlusion, drugs, and chemicals, environmental factors, and interactions with systemic diseases such as diabetes and cardiovascular diseases.

As systematic mechanisms underlying periodontitis are complex, it remains difficult to elucidate relationships and interactions between the multiple risk factors through studies on individual molecules only. Analyses based on pathways and networks are required in order to elucidate relationships and interactions from ‘omics data’, such as genomics, transcriptomics, proteomics, and metabolomics observed in molecular pathways that are involved in the pathogenesis and progression of periodontitis [28]. Actually, gene expression data from periodontal tissue has allowed the partial elucidation of such molecular pathways [29]. However, previous analyses of detecting disease-specific processes have not been so successful. This may be due to the complexity of periodontitis, as well as to the way results from omics analyses have been semantically interpreted. In omics analysis, GO is generally used in annotations of the data; however, few processes specific to periodontitis are included in GO. This was our motivation to enhance the GO by using a periodontitis-specific extension.

Kornman proposed a systems model to link the clinical presentation of periodontitis to underlying molecular knowledge and thus better clarify the pathogenesis of periodontal diseases [30].

In the past decade, molecular details have been elucidated in periodontitis comparison with other osteoimmunology-based diseases such as rheumatoid arthritis [31]. Osteoimmunology is an interdisciplinary science investigating the interplay between the skeletal and the immune systems. The main contributors to osteoimmunology are bone effector cells such as osteoclasts or osteoblasts, and immune cells, particularly lymphocytes and monocytes [32]. Osteoimmunology has now become one of the most prominent research areas in clinical biology, and periodontitis is considered to be a good model for the study of the common mechanisms in osteoimmunology and the progression of target diseases. Several studies relating periodontitis to osteoimmunology have recently been reported [33, 34].

In this paper, we report on Periodontitis-Ontology (PeriO), an ontology we developed for periodontitis. This ontology covers and formally describes a variety of entities that stand in relation to periodontitis. PeriO describes relationships between molecular mechanisms of inner processes in periodontitis and pathogenesis and progression in a clinical view of the disease, as well as relationships between molecular influences of drugs and environmental molecules and clinical medications and treatments.

Content integrated into our PeriO includes the following: 1) functional classification of bacterial molecules in periodontal lesions; 2) interactions between periodontitis and other systemic diseases; 3) environmental chemicals affecting periodontitis; and 4) processes of medical treatments for and the molecular pathogenesis of periodontitis.

## Methods

PeriO is based on our previous development of ontology, which specified the bone resorption response induced by periodontitis [35]. Bone resorption is one part of a larger process in the onset and progression of periodontitis; the entire process is composed of many biological processes and clinical actions. We systematized the entire process in this study as PeriO.

### Extraction of terms relating to periodontitis

We retrieved review articles for periodontitis by from the PubMed using keywords of ‘periodontitis, biology, human and review’ on April 30, 2014. In order to collect mentions of molecules and cells participating in processes we formalized in PeriO, we investigated PubMed abstracts reporting on the individual process concepts. References to concepts were manually extracted from review articles and abstracts by experts in periodontology (authors of this manuscript) when the terms were related to its onset and progression.

### Ontology Construction

We developed PeriO using OBO-Edit version 2.3-beta5. OBO-Edit is an open source, platform-independent ontology editor developed and maintained by the Gene Ontology Consortium. OBO-Edit is a tool for domain experts to browse, search, and edit ontologies. OBO-Edit continues to undergo active development in response to the needs of its users [36, 37].

### Comparison of PeriO classes with GO-BP

We investigated matching between classes of PeriO and that of GO-BP. Every class of PeriO was manually examined against classes of GO-BP as to whether an equivalent class was found in GO-BP. AMIGO (http://amigo.geneontology.org/amigo) was employed to search GO-BP.

## Results

### Extraction of terms that were characteristic in descriptions of periodontitis

We identified 194 review articles from PubMed on the development, progression, and treatment of periodontitis. From the collected articles, we excluded those reporting on apical periodontitis because it is a distinct disease from general periodontitis in terms of molecular pathology.

A total of 101 articles were available as full texts, 79 articles were available as abstracts only, and 14 articles were unavailable (Table 1). All 14 unavailable articles were written in languages other than English, with the only useful information being the titles.

**Table 1:** The full texts and abstracts used in this study

Review articles	No. of articles
Full texts	101
Abstracts only	79
Unavailable	14
Total	194

The 194 articles were published in a total of 50 journals, the five most frequent of which were *Journal of Periodontology* (21 articles), *Periodontology 2000* (16 articles), *Journal of Clinical Periodontology* (16 articles), *Journal of Dental Research* (12 articles) and *Annals of periodontology* (8 articles) (Additional file 1).

In addition, we collected abstracts related to the medical treatment and molecular pathogenesis of periodontitis. We found 22 abstracts on ‘medical treatment for oral biofilm’, 229 on ‘medical treatment for inflammation’, 58 on ‘medical treatment for pathological bone loss’, 126 on ‘laboratory test for periodontitis’ in the process of medical treatment for periodontitis, 117 on ‘formation of oral biofilm’, 477 on ‘inflammation in gingiva’, 137 on ‘invasion of bacteria’, and 225 on ‘pathological bone resorption’ in the process of molecular pathogenesis of periodontitis.

We manually extracted 1,347 characteristic terms of periodontitis from the review articles and abstracts retrieved from PubMed. We systematized the extracted terms and developed an ontology called ‘PeriO’. The distribution of classes according to top categorization in PeriO is shown in Table 2.

**Table 2:** Distribution of the terms according to the classes in PeriO

Top categorization in PeriO			No. of subsumed classes
object	cell		96
	cell part		4
	extended organism		81
	living organism		71
	medical material		13
	molecule	bacterial molecule	72
		chemical drug	227
		human molecule	508
object aggregate	molecule aggregate		27
	organism aggregate		2
realized entity	disposition	disease	109
process	bodily process	molecular pathogenesis of periodontitis	79
		physical process	2
	treatment	medical treatment for periodontitis	56
Total			1,347

### Structure of PeriO

#### Top categories in is-a hierarchy

We categorized concepts denoted by the extracted terms on molecular pathology and periodontal processes into a ‘is-a’ hierarchy. Our categorization refers to Basic Formal Ontology (BFO) [38] and OBO Relation Ontology (OBO-RO) [39]. BFO is a top-level ontology that serves as a domain-neutral framework for the development of lower level ontologies in many specialist disciplines, above all in biology and medicine. BFO gives a formal account of the distinctions between: (a) universal and particular; (b) continuant and occurrent; (c) dependent and independent; and (d) formal and material [38]. OBO-RO provides consistent and unambiguous formal definitions of relations used in biomedical ontologies. It focuses on definitions of general-purpose relations that can be employed, in principle, in all biological ontologies, including ‘*is_a’, ‘part_of’, ‘located_in’, ‘contained_in’, ‘adjacent_to’, ‘transformation_of’, ‘derives_from’, ‘preceded_by’, ‘has_participant’*, and ‘*has_agent’* [39].

**Fig. 1:**
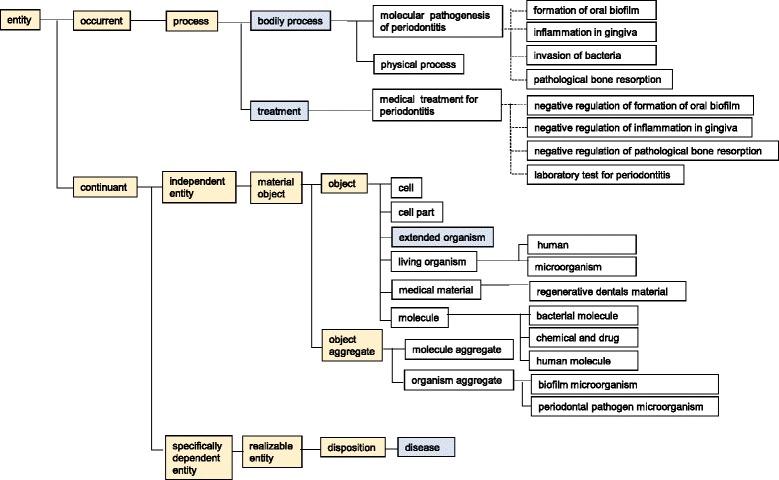
A hierarchy of upper categories in our ontology for molecular pathology in periodontitis. This figure shows upper categories from the first to the seventh level of PeriO. It shows all the classes in the first, second, third, fourth, and fifth levels, while only representative classes are shown in the sixth and seventh levels. Edges in solid lines indicate ‘*is_a*’ relations. Edges in broken lines indicate *‘has_part’* relations. Classes in red were inherited from BFO. Classes in blue were inherited from OGMS

Top categories of PeriO were inherited from BFO and OGMS [40] (Figure 1). This ontology starts with ‘entity’, which is divided into ‘occurrent’ and ‘continuant’. ‘Occurrent’ subsumes ‘process’, which subsumes ‘molecular pathogenesis of periodontitis’ and ‘medical treatment for periodontitis’. ‘Continuant’ subsumes ‘independent entity’ and ‘specifically dependent entity’; the former subsumes ‘cell’, ‘cell part’, ‘extended organism’, ‘living organism’, ‘medical material’, ‘molecule’, ‘molecule aggregate’, and ‘organism aggregate’,. The latter subsumes ‘disposition’, which subsumes ‘disease’. Edges in Fig. 1 indicate ‘*is_a*’ or *‘has_part’* relations. All arguments in PeriO are embraced in the domain of the molecular pathology of periodontitis.

#### Properties characterize classes

In PeriO, the classes are linked by relations such as *‘is_a’* and *‘has_part’*. We follow the definition in the OBO-RO [39]: *‘is_a’* (OBO-RO:0000001) and *‘has_part’* (OBO-RO:0000003). The classes are characterized by 1) PubMed IDs of reference papers reporting on characterization of the term; 2) synonymous terms from reference papers; and 3) IDs of corresponding entries in existing biomedical ontologies. Our hierarchical conceptual tree consists of both ‘*is_a’* and *‘has*_*part’* relationships and allows multiple parenthood in both taxonomic and partonomic hierarchies. We tried to make PeriO directly reflect background knowledge of experts on periodontology as much as possible, so as to make the ontology familiar to the domain experts and facilitate the application of the ontology in periodontitis research. As the success of GO shows, ontologies are useful in omics research with both taxonomic and partonomic hierarchies.

The interoperability of existing biomedical ontologies is an important property of the characterization of the concepts. The terms are characterized by corresponding ontologies as follows: 1) GO for the class ‘process’; 2) Chemical Entities of Biological Interest Ontology (ChEBI) [41] for the class ‘molecule’ and ‘medical material’; 3) Cell Ontology (CL) [42] for the class ‘cell’; 4) Foundational Model of Anatomy Ontology (FMA) [43] for the class ‘organism part’; and 5) Experimental Factor Ontology (EFO) [44] for the class ‘anatomy’, ‘disease’ and ‘chemical compounds’.

#### Properties particularly characterize the class ‘process’

**Fig. 2:**
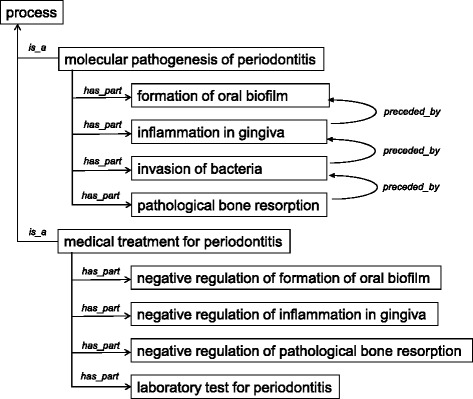
Process characterizing periodontitis. ‘Process’ subsumes two counter concepts of ‘molecular pathogenesis of periodontitis’, a process of development of periodontitis, and ‘medical treatment for periodontitis’, a process of recovery from periodontitis. Individual processes are divided into sub-processes by *‘has_part*’ relationships. Please note *‘preceded by’* relations (causal relationships) characterize the sub-processes of ‘molecular pathogenesis of periodontitis’, which indicates essential features following a specified event in time causing a disease state as a consequence in periodontitis

In PeriO, we regard ‘process’ as the most important class for describing molecular mechanisms of disease development in periodontitis (Fig. 2). The ‘process’ class was modeled referring to BFO [38] and RO [39], and specified by additional relations such as *‘preceded_by’*, ‘*located_in*’, ‘*has_participant*’, ‘*has*_*active_agent’* and ‘*has*_*suppressive_agent*’.

1. ‘*preceded_by*’ (OBO-RO:0000017): is a process preceded by the process by causal relation in a cascade of molecular processes. Both the processes should belong to ‘process’ class.
2. ‘*located_in*’ (OBO-RO:0000008): is a place where the process occurs. The place should belong to ‘extended organism’ or ‘cell’ class.
3. ‘*has_participant*’ (OBO-RO:0000019): is an object that participates in the process. The object should belong to ‘cell’ or ‘living organism’ class.
4. *‘has_active_agent’* (subclass of OBO-RO:0000021): is an object that participates in the process and causally activates the process. The object should belong to ‘molecule’ class including ‘human molecule’ and ‘bacterial molecule’ class.
5. *‘has_suppressive_agent’* (subclass of OBO-RO:0000021): is an object that participates in the process and causally suppresses the process. The object should belong to ‘molecule’ class including ‘human molecule’ and ‘bacterial molecule’ class.

We followed the relations defined in OBO-RO. As ‘*has*_*active_agent*’ and ‘*has*_*suppressive_agent*’ were derived relations from OBO-RO, we defined them as follows:

A. *‘has_active_agent’*: is a subclass of ‘*has*_*agent*’ (OBO-RO:0000021) representing a relation whose consequence is activation of a process in which the agent participates.
B. ‘*has*_*suppresive_agent*’: is a subclass of ‘*has*_*agent*’ (OBO-RO:0000021) representing a relation whose consequence is suppression of a process in which the agent participates.

**Fig. 3:**
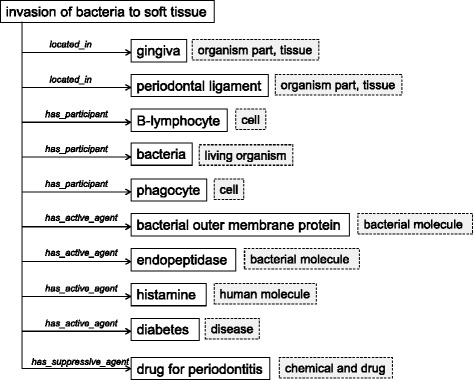
Characterization of a process concept by relations to concepts under ‘continuant’ class. We take ‘invasion of bacteria to soft tissue’ as an example. The process is characterized by 10 kinds of concepts subsumed by ‘continuant’ class with relations such as ‘*located_in*’, ‘*has_participant*’, ‘*has_active_agent*’, *‘has_*suppressive_agent’. These relations illustrate that the process of ‘invasion of bacteria to soft tissue’ occurs in gingiva and periodontal ligaments containing B-lymphocytes, phagocytes, and microorganisms, actively regulated by bacterial outer membrane proteins, endopeptidase, and histamine, as well as systemic disease of diabetes, and suppressed by drugs for periodontitis. Every relation is annotated by a reference paper reporting on a reason of the relation

Fig. 3 shows how these relations specify a ‘process’, taking ‘invasion of bacteria to soft tissue’ as an example.

As shown in GO-BP, relations between processes and biological entities play significant roles in the computational analysis of omics data. We specified the relations into the following three kinds: *‘has_active_agent’*; *‘has_suppressive_agent’*; and *‘has_participant’.* While *‘has_active_agent’* and *‘has_suppressive_agent’* relations are accounted by the class ‘molecule’ including ‘human molecule’ and ‘bacterial molecule’, *‘has_participant’* relations are accounted by the class ‘cell’ and ‘living organism’ including ‘bacterium’. That reflects background knowledge of domain experts on periodontology:

1. The domain experts have major interests in molecules that can regulate (activate or suppress) the ‘process’ because they explore a molecule that can change the process of malignant into benign consequence. The molecules and their regulation (activation or suppression) provide clues for development of drugs for periodontitis.
2. The experts regard processes in periodontitis as subsequent interactions among cells and bacteria mediated by active or suppressive interventions of biological molecules. In the view of BFO/RO, participants are played by cells and living organisms, which discriminates PeriO from GO-BP, in which participants are played by molecules (gene products).

### Representation of process in PeriO, in comparison with GO

#### Representation of inner biological processes in periodontitis, in comparison with GO

PeriO mainly targets the collection and classification of biological processes in the pathogenesis and progression of periodontitis. As GO-BP is frequently used for annotation of omics data, formalized knowledge of inner processes of biological phenomena provides us with a guide to mine useful information from massive data such as genome-wide gene expression profiles. The popularity comes from the fact that biologists consider biological processes as manifestations of causal relationships between biological molecules that result in observable biological phenomena, even though the details of their causal relationships have not been clearly elucidated.

GO-BP includes almost all of the processes known in the research domain of biology; however, the ontology does not include the class ‘periodontitis’. Therefore, we investigated whether the classes of GO-BP match those we collected for specification of periodontitis. We investigated differences between GO-BP and PeriO. Additional file 2 and Additional file 3 show the results of mapping between the classes of both ontologies. We found that most of the classes for processes in PeriO could be mapped to the classes of GO-BP, in a way of 1) equivalent class, 2) class with *‘is_a’* relationship, or 3) class with *‘has_part’* relationship. For example, ‘multinucleation of osteoclast’ (in PeriO) was linked to ‘multinuclear osteoclast differentiation’ (GO:0072674) by an ‘equivalent’ relationship, ‘production of M-CSF’ (in PeriO) was linked to ‘production of molecular mediator involved in inflammatory response’ (GO:0002532) by an *‘is_a’* relationship, and ‘survival of osteoclast’ (in PeriO) was linked to ‘osteoclast development’ (GO: 0036035) by a *‘has_part’* relationship.

**Fig. 4:**
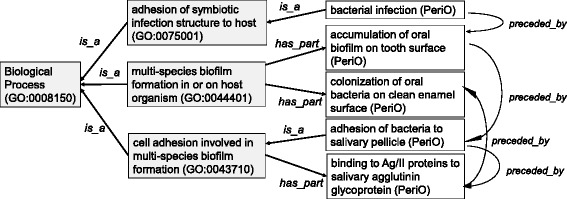
Comparison between PeriO and Biological Process ontology in GO (GO-BP). The classes of PeriO can be mapped to the classes of Biological Process in GO (GO-BP) with ‘*is_a*’ or *’has*_*part*’ relationships. This indicates that PeriO defines more specific concepts specific to tissues and cells as well as contexts of periodontitis than GO-BP. The classes in PeriO are characterized by causal (*‘preceded by’*) relationships in series, which represents the significance of secession among processes and of consequence of the successive processes resulting in a disease-state

In the mapping results, we should pay attention to the fact that we needed *‘is_a’* or *‘has_part’* relations for the mappings. This fact indicated that PeriO included more specific concepts than GO-BP. This specificity is illustrated in Fig. 4. PeriO describes the biological processes observed in certain cells and genes specific to periodontitis, i.e., the cells migrated into the periodontal lesions and the genes activated in the periodontal lesions. Sometimes such specific processes are named very differently in GO-BP. This is particularly the case in subclasses of ‘bone remodeling’ and ‘negative regulation of bone remodeling’, both of which are most characteristic processes of periodontal pathology. PeriO specifies ‘degradation of bone matrix in gingival crevicular fluid’, which is connected to the BP class ‘bone remodeling’ in BP via the existentially qualified relation (*‘has*_*part’*). PeriO specifies ‘loss of balance between bone resorption and bone formation’ and ‘osteoclastic bone resorption exceeds osteoblastic bone formation’, both of which are connected to the BP class ‘negative regulation of bone remodeling’ via the existentially qualified relation (*‘has*_*part*’). These classes of PeriO identify minute details observed specifically in periodontal lesions and periodontal progression. These concepts can compose a subset of classes added as descendants of GO-BP leaf nodes, which can enhance GO-BP by a periodontitis-specific extension. We believe that such a subset of disease specific concepts will be useful when analyzing data with more disease specificity and obtaining outcomes with more clinical validity.

We also found a certain difference between PeriO and GO-BP in the structure of relationships between the concepts for processes. While GO-BP defines *‘is_a’* and/or *‘part_of’* relationships among the process concepts, PeriO primarily defines causal relationships (‘*preceded_by*’) between the process concepts (Fig. 4). The structure based on causality is familiar to the domain experts including dentists who consider periodontitis as a conclusion of a series of processes that occur in periodontal lesions. Therefore, the domain experts regard the causal relationships in pathogenesis and progression of periodontitis as most important. In the OBO-Editor (OBO-Edit), the causal relationships cannot be directly represented in tree style in its ontology viewer, so we provisionally numbered the classes in sequence in order to represent *‘precedent_by’* relationships among the concepts for processes (*e.g.*, ‘1 formation of oral biofilm’ and ‘2 inflammation in gingiva’), which is regarded as more important for reasoning than ‘*is_a’* in the periodontal research domain.

In conclusion, we found peculiarities of PeriO existed in 1) granularity and context dependency of both the conceptualizations, and 2) causality intrinsic to the pathological processes: 1) PeriO defines more specific concepts that can be inserted under the leaf concepts of GO-BP, and 2) GO-BP uses *‘is_a’* and/or *‘part_of’* relationships, while PeriO primarily consists of causal relationships in case of formalization of process concepts. We believe this can be common to other diseases when comparing processes in disease against GO-BP.

#### Representation of medical treatment for periodontitis, in comparison with GO

Our PeriO includes concepts on ‘medical treatment for periodontitis’ in alignment with ‘molecular pathogenesis of periodontitis’. We considered that PeriO should specify biological processes not only for development of, but also of recovery from disease.

Sub-categories of ‘medical treatment for periodontitis’ were counterbalanced with the sub-categories of ‘molecular pathogenesis of periodontitis’, as exemplified in comparison with the top three categories of both classes (Fig. 2):

molecular pathogenesis of periodontitis

- *has_part* formation of oral biofilm
- *has_part* inflammation in gingiva
- *has_part* pathological bone resorption

medical treatment for periodontitis

- *has_part* negative regulation of formation of oral biofilm
- *has_part* negative regulation of inflammation in gingiva
- *has_part* negative regulation of pathological bone resorption.

The mirror image indicated that medical treatments were taken to individual processes in the development into periodontitis, which could be reasonable considering that medical treatments using modern technology target the biological processes observed in the progress of the disease.

**Fig. 5:**
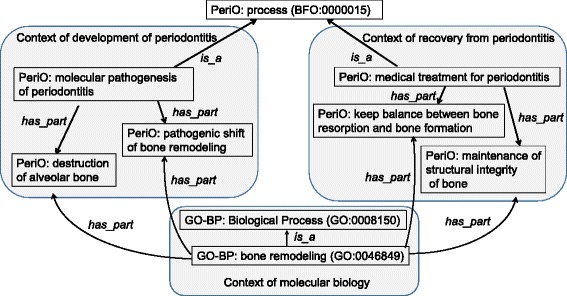
Relationships in Process between PeriO and GO. This figure illustrates relationships between classes of ‘medical treatment for periodontitis’, ‘molecular pathogenesis of periodontitis’, and GO-BP. ‘Bone remodeling’ (GO:0046849) is referred not only by ‘pathogenic shift of bone remodeling’ and ‘destruction of alveolar bone’, but also by ‘keep balance between bone resorption and bone formation’ and ‘maintenance of structural integrity of bone’

In order to investigate the symmetrical relationships between ‘medical treatment for periodontitis’ and ‘molecular pathogenesis of periodontitis’, we again mapped the terms to GO-BP. As Additional file 3 shows, most of all the classes under ‘medical treatment for periodontitis’ were mapped to the classes in GO-BP, with the exception of ‘laboratory tests for periodontitis’. When mapping to GO-BP, both the corresponding classes of ‘medical treatment for periodontitis’ and ‘molecular pathogenesis of periodontitis’ linked to the same or closely related GO-BP classes. For example (Fig. 5), ‘bone remodeling’ (GO:0046849) was referred not only by ‘pathogenic shift of bone remodeling’ and ‘destruction of alveolar bone’, but also by ‘keep balance between bone resorption and bone formation’ and ‘maintenance of structural integrity of bone’. The same was true for ‘osteoblast development’ (GO:0002076).

Fig. 5 illustrates relationships between classes of ‘medical treatment for periodontitis’, ‘molecular pathogenesis of periodontitis’, and GO-BP. The classes in GO-BP represented something neutral without premising clinical contexts. When premising clinical contexts, the GO-BP classes should be specialized in features of the development into or recovery from a certain disease state such as periodontitis. GO-BP defines the biological foundation of a certain biological process observed in a ubiquitous context, while PeriO defines roles of the biological process in contexts of either the development into or the recovery from periodontitis.

### Specific classes to periodontitis

PeriO includes specific classes not included in MeSH, ICD, GO, or KEGG. ‘Bacterial molecule’ (‘*is_a*’ ‘molecule’) and ‘regenerative dental material’ (‘*is_a*’ ‘medical material’) are the specific classes. These represent features specific to periodontitis.

‘Bacterial molecule’ represents a protein produced by a bacterium. Entities that belong to ‘bacterial molecule’ are classified from a viewpoint of their influences upon the generation and progression of periodontitis. ‘Bacterial molecule’ is grouped into ‘exotoxin’ and ‘endotoxin’ according to toxic properties against human cells. After the first grouping, the molecules are categorized according to their functions in periodontal pathogenesis.

‘Regenerative dental material’ represents material used in clinical treatments for alveolar bone and gingival regeneration. ‘Regenerative dental material’ is related to ‘periodontal regeneration therapy’ by *‘has_agent’*.

### Labeling for identical names of different functionality or species

The ‘chemical and drug’ class subsumes several metabolic products used as drugs. Examples are ‘estrogen’ and ‘histamine’. In order to distinguish these drugs from biological metabolic products, PeriO uses the name of a drug combined with the name of its functionality:

- ‘estrogen [human]’ *is_a* ‘human molecule’
- ‘estrogen [drug]’ *is_a* ‘drug’

The ‘bacterial molecule’ class subsumes homologous genes to humans. Similar naming is employed in order to discriminate the genes. For example, ‘collagenase’ is a protein produced by both humans and bacteria. In this case, PeriO uses a name of a gene combined with the name of its species:

- ‘collagenase [human]’ *is_a ‘*human molecule’
- ‘collagenase [bacteria]’ *is_a* ‘bacterial molecule’.

### Ontological specification for recognition of clinical dentists and periodontal researchers

**Fig. 6:**
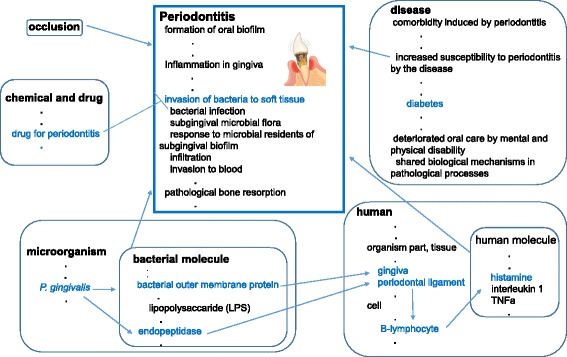
Clinical interpretation of process ‘invasion bacteria to soft tissue’ in the molecular pathogenesis of periodontitis. Clinical dentists and periodontal researchers assigned pathological processes in PeriO to clinical pathways based on specific knowledge

PeriO uses several networks among molecules based on basic textbooks in order to explicate knowledge implicit for clinical dentists and periodontal researchers (Fig. 6). This pathway indicates interactions between bacteria and periodontal tissue and their induced cellular causal networks that result in periodontitis.

**Fig. 7:**
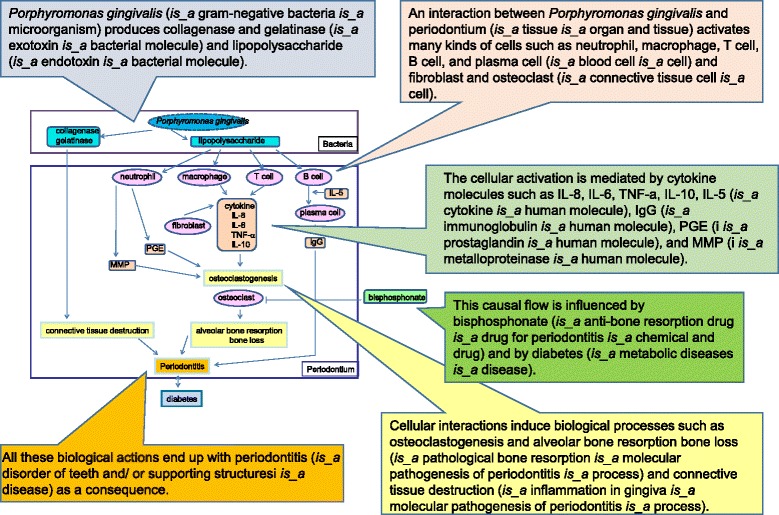
Ontological specification for recognition of clinical dentists and periodontal researchers. This figure shows a simplified pathway for the onset and progression of periodontitis, which many clinical dentists and periodontal researchers agree on. All of the biochemical and clinical entities are specified in PeriO. Entities in this figure are shown in colours according to class

Fig. 7 shows the interactions between the cells that induce pathological processes such as ‘osteoclastogenesis and alveolar bone resorption’ and ‘bone loss’ (*‘is_a’* ‘molecular pathogenesis of periodontitis’ *‘is_a’* ‘pathological bone resorption’ *‘is_a’* ‘process’) and connective tissue destruction responses result in ‘periodontitis’ (*‘is_a*’ ‘periodontal disease’ *‘is_a’* ‘disorder of teeth and/or supporting structures’ *‘is_a’* ‘disorder of digestive system’ *‘is_a’* ‘disease’) as a consequence. This causal flow is influenced by ‘bisphosphonate’ (*‘is_a’* ‘anti-bone resorption drug’ *‘is_a’* ‘drug for periodontitis’ *‘is_a’ ‘*chemical and drug’) and by ‘diabetes’ (*‘is_a’* ‘metabolic disease’ *‘is_a’* ‘disease’). PeriO specifies all the entities of this pathway; therefore, PeriO provides implicit agreements.

## Discussion

### Limitation of PeriO and future research

Although we investigated 101 review articles of full texts and 1,391 abstracts in building our ontology, we do not consider the current PeriO to be complete. Additional knowledge should be collected from more original articles; however, due to the rapid accumulation of publications in the field and an interdisciplinary range of knowledge spreading over the clinical, medical, biological, chemical, and bacterial disciplines, manual collection of new knowledge is resource intensive and thereby quite difficult. We consider this ontology as a core resource of knowledge for future development of computational tools that enable us to automatically extract classes from the literature. A natural language processing tool is under development for the suggestion of new ontology content on the basis of PeriO.

### Application to other complex diseases

There is an obvious need for systematic integration of information in multiple levels of substances, cells, organs, and clinical outcomes in periodontology [41, 45]. Currently, many web sites maintain highly elaborated structured knowledge for diseases like Alzheimer’s disease [46], Crohn's Disease [47], and asthma [48], as well as many other diseases. Specific ontologies must be developed for specific diseases because every disease includes knowledge specific to that disease. PeriO, with its upper categorization and relations between the higher classes, can be reused in ontological development for other diseases.

### Relationship between periodontitis and systemic diseases

**Fig. 8:**
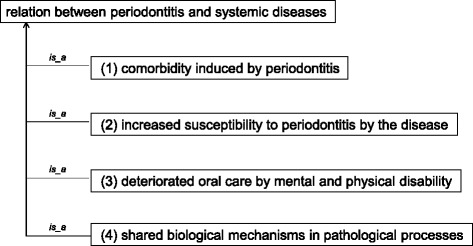
Relation between periodontitis and systemic disease. Relations between periodontitis and systemic diseases are divided into four categories

Although periodontal lesions are located in the oral cavity, they may induce other systemic diseases such as cardiovascular diseases and diabetes. Actually, progression of periodontitis interdepends on various diseases with mediation mainly by osteoimmune responses. Medical treatments for periodontitis can significantly affect the progression of many systemic diseases. We collected mentions of systemic diseases related to periodontitis from the articles listed in Additional file 1 and classified them into the following seven categories based on ICD10: ‘disorder by body site’; ‘disorder of fetus or newborn’; ‘disorder of immune function’; ‘infectious disease’; ‘metabolic disease’; ‘neoplastic disease’; and ‘syndrome’. We investigated relationships between periodontitis and the systemic diseases and found the following four patterns (Fig. 8):

1. *‘comorbidity induced by periodontitis’*, which relates such diseases as ‘coronary heart artheriosclerosis’ and ‘low birth weight infant’ with ‘periodontitis’.
2. *‘increased susceptibility to periodontitis by the disease’*, which relates such diseases as ‘type I diabetes’ and ‘Papillon-Lefevre syndrome’ with ‘periodontitis’.
3. *‘deteriorated oral care by mental and physical disability’*, which relates such diseases as ‘Alzheimer’s disease’ with ‘periodontitis’.
4. *‘shared biological mechanisms in pathological processes’*, which relates such diseases as ‘rheumatism’ and ‘collagen-induced arthritis’ with ‘periodontitis’.

Although this is an early result of specification for interdependency among diseases, these relations are expected to be helpful in the comprehensive collection and analysis of the interdependence between different diseases in larger-scale studies in the future.

We plan on logically formalizing these relations on the basis of RO in our next study.

## Conclusions

In PeriO, we explicated all types of entities contributing to the development, progression, and treatment of periodontitis. PeriO explicates relations between processes and other entities with reference to BFO and RO, which accounts for participants in the processes realized by biological objects such as molecules and cells. Comparing the ‘process’ class with GO-BP, we found the following differences in conceptualization of both the ontologies:1) PeriO defines more specific concepts than GO-BP; these concepts can be added as descendants of GO-BP leaf nodes; and 2) GO-BP uses *‘is_a’* and/or *‘part_of’* relationships, while PeriO primarily consists of causal relationships; this indicates the intrinsic causality in conceptualization of the processes in periodontitis, reflecting the domain knowledge where a consequence in the causal relationships is a primal interest by the domain experts (periodontal doctors and researchers). We believe this can be common to other diseases when comparing processes in diseases with GO-BP.

PeriO can be a model for ontological integration of knowledge for other multifactorial diseases like chronic inflammation in different organs and disorders of immune systems. Formalization of processes brings opportunities for clinical dentists and periodontal researchers to elucidate pathways and mechanisms of the pathogenesis and progression of periodontitis and clinical treatment. As PeriO was founded on extracted knowledge, more knowledge should be collected from original articles using text mining techniques and employing PeriO as a reference ontology.

PeriO is available at http://bio-omix.tmd.ac.jp/periodontitis/ and Additional file 4 in OBO format.

## Additional files

Additional file 1:
**This file contains references of the reviews used in this study.**
Additional file 2:
**This Table contains all the mapping results of the classes in processes of molecular pathogenesis of periodontitis between PeriO and GO-BP.**
Additional file 3:
**This table contains all the mapping results of the classes in processes of medical treatment for periodontitis between PeriO and GO-BP.**
Additional file 4:
**PeriO in OBO format version 2.3-beta 5.**

## Abbreviations

BFO: Basic Formal Ontology
ChEBI: Chemical Entities of Biological Interest Ontology
CL: Cell Ontology
DO: Disease Ontology
DOID: Human Disease Ontology
EFO: Experimental Factor Ontology
ENCODE: Encyclopedia of DNA Elements
FMA: Foundational Model of Anatomy Ontology
GO: Gene Ontology
GO-BP: Gene Ontology Biological Process
ICD: International Classification of Diseases
IDO: Infectious Disease Ontology
IgG: Immunoglobulin G
IL-10: Interleukin-10
IL-5: Interleukin-5
IL-6: Interleukin-6
IL-8: Interleukin-8
KEGG: Kyoto Encyclopedia of Genes and Genomes
M-CSF: Macrophage-Colony Stimulating Factor
MeSH: Medical Subject Headings
MMP: matrix metalloproteinase
NIH: National Institutes of Health
OBO: Open Biological and Biomedical Ontologies
OBO-Edit: Open Biological and Biomedical Ontology Editor
OBO-RO: Open Biological and Biomedical ontologies-Relation Ontology
OGM: Ontology for General Medical Science
PGE: Prostaglandin
SNP: Single Nucleotide Polymorphism
TNF-alpha: Tumor Necrosis Factor-alpha
UMLS: Unified Medical Language System
