# Supplementary material for: Development of an Ontology for Periodontitis
Source: J Biomed Semantics. 2015 Jul 1;6:30. doi: 10.1186/s13326-015-0028-y (PMC4488034; doi:10.1186/s13326-015-0028-y)
Supplement: Additional file 1: — This file contains references of the reviews used in this study. [file 13326_2015_28_MOESM1_ESM.pdf]

Additional Table 1-1 Classes of "molecular pathogenesis of periodontitis" in PeriO compared with classes in GO-BP.

first category	second category	Class of PeriO	relation	Class of GO-BP	OBO ID
formation of oral biofilm			equivalent	multi-species biofilm formation	GO:0044399
	early stage of biofilm formation		has part	multi-species biofilm formation	GO:0044399
		bacterial infection	is a	adhesion of symbiotic infection structure to host	GO:0075001
		accumulation of oral biofilm on tooth surface	is a	multi-species biofilm formation in or on host organism	GO:0044401
		adhesion of bacteria to salivary pellicle	is a	cell adhesion involved in multi-species biofilm formation	GO:0043710
		binding to Ag/II proteins to salivary agglutinin glycoprotein	has part	cell adhesion involved in multi-species biofilm formation	GO:0043710
		colonization of oral bacteria on clean enamel surface	has part	multi-species biofilm formation in or on host organism	GO:0044401
		coadhesion of bacteria from saliva	has part	formation of infection structure on or near host	GO:0075015
		inclusion of extracellular DNA as integral component of binding	has part	multi-species biofilm formation in or on host organism	GO:0044401
		production of hydrogen ions by aciduric and acidorganic organisms	has part	organic acid metabolic process	GO:0006082
		Production of organic acid from sugars	has part	organic acid metabolic process	GO:0006082
	growth of biofilm		equivalent	multi-species biofilm formation	GO:0044399
		conversion of sucrose into extracellular glucagon and fructan polymers	has part	sucrose metabolism	GO:0005985
		degradation of mucins and other salivary components	has part	glycoprotein catabolic process	GO:0006516
		development of microenvironment with biofilm	has part	multi-species biofilm formation	GO:0044399
		incorporation of acidogenic organisms into biofilm	has part	multi-species biofilm formation	GO:0044399
		maturation of biofilm	has part	multi-species biofilm formation	GO:0044399
		population change from commensal bacteria	has part	competition with other organism	GO:0044402
inflammation in gingiva			is a	inflammatory response	GO:0006954
	endocytosis		equivalent	endocytosis	GO:0006897
		chemotaxis	equivalent	leukocyte chemotaxis involved in inflammatory response	GO:0002232
		coagulation	equivalent	coagulation	GO:0050817
		fibrosis	has part	connective tissue replacement involved in inflammatory response	GO:0002248
		phagocytosis	equivalent	phagocytosis	GO:0006909
	destruction of connective tissue		NA	NA	NA
		increase of concentration of inflammatory mediators in gingival tissue	has part	production of molecular mediator involved in inflammatory response	GO:0002532
		increase of concentration of cathepsin K in gingival fluid	is a	gene expression involved in extracellular matrix organization	GO:1901148
		penetration of inflammatory mediators into gingival tissue	has part	translocation of molecules into other organism involved in symbiotic interaction	GO:0051836
		activation of MMP in osteogenic cells	has part	extracellular matrix disassembly	GO:0022617
		increased expression of RANKL	is a	positive regulation of tumor necrosis factor (ligand) superfamily member 11	GO:2000309
		binding of RANKL to RANK on surface of preosteoblast	is a	RANKL-mediated signaling pathway	GO:0071847
		increased expression of RANKL on immune cell	is a	positive regulation of tumor necrosis factor (ligand) superfamily member 11	GO:2000309
		increased expression of RANKL on muskoskelatal cell	is a	positive regulation of tumor necrosis factor (ligand) superfamily member 11	GO:2000309
		increased expression of RANKL on osteogenic cell	is a	positive regulation of tumor necrosis factor (ligand) superfamily member 11	GO:2000309
	production of cytokines and inflammatory		is a	production of molecular mediator involved in inflammatory response	GO:0002532
invasion of bacteria			equivalent	entry of bacterium into host cell	GO:0035635
	invasion of bacteria to soft tissue		has part	entry of bacterium into host cell	GO:0035635
		bacterial infection	is a	adhesion of symbiotic infection structure to host	GO:0075001
		subgingival microbial flora	is a	multi-species biofilm formation in or on host organism	GO:0044401
		response to microbial residents of subgingival biofilm	has part	multi-species biofilm formation in or on host organism	GO:0044401
		infiltration to soft tissue	has part	entry of bacterium into host cell	GO:0035635
	invasion of bacteria to cardio-vascular system		has part	entry of bacterium into host cell	GO:0035635
		formation of atherosclerotic plaque	has part	adhesion of symbiotic infection structure to host	GO:0075001
pathological bone resorption			equivalent	bone resorption	GO:0045453
	inflammation in alveolar bone		is a	production of molecular mediator involved in inflammatory response	GO:0002532
		aggregation of T-cells and B-cells in gingival soft tissue	is a	leukocyte migration involved in inflammatory response	GO:0002523
	osteoclast differentiation		equivalent	osteoclast differentiation	GO:0030316
		differentiation of monocyte/macrophage into osteoclast	is a	monocyte differentiation, macrophage differentiation	GO:0030224, GO:0030225
		multinucleation of osteoclast	equivalent	multinuclear osteoclast differentiation	GO:0072674 :
		production of M-CSF	is a	production of molecular mediator involved in inflammatory response	GO:0002532
		production of M-CSF by osteoblast	is a	production of molecular mediator involved in inflammatory response	GO:0002533
		production of M-CSF by bone marrow stromal cell	is a	production of molecular mediator involved in inflammatory response	GO:0002534
	osteoclastogenesis		is a	osteoclast differentiation	GO:0030316
		production of M-CSF	is a	production of molecular mediator involved in inflammatory response	GO:0002532
		proliferation preosteoclast	has part	osteoclast proliferation	GO:0002158
		survival of preosteoclast	has part	osteoclast development	GO:0036035
	pathogenic shift of bone remodeling		has part	negative regulation of bone remodeling	GO:0046851
		loss of balance between bone resorption and bone formation	has part	negative regulation of bone remodeling	GO:0046851
		osteoclastic bone resorption exceeds osteoblastic bone formation	has part	negative regulation of bone remodeling	GO:0046851
	destruction of alveolar bone		has part	bone resorption	GO:0045453
		degradation of bone matrix in gingival crevicular fluid	has part	bone remodeling	GO:0046849
		erosion of bone	has part	bone resorption	GO:0045453
		increased RANKL/OPG ratio	is a	positive regulation of gene expression involved in extracellular matrix organization	GO:1901313
		periodontal bone resorption	is a	bone resorption	GO:0045453

NA: Not Available
