# Supplementary material for: Development of an Ontology for Periodontitis
Source: J Biomed Semantics. 2015 Jul 1;6:30. doi: 10.1186/s13326-015-0028-y (PMC4488034; doi:10.1186/s13326-015-0028-y)
Supplement: Additional file 2: — This Table contains all the mapping results of the classes in processes of molecular pathogenesis of periodontitis between PeriO and GO-BP. [file 13326_2015_28_MOESM2_ESM.pdf]

Additional Table 1-2 Classes of "medical treatment" in PeriO compared with classes in GO-BP.

first category	second category	Class of PeriO	relation	Class of GO-BP	OBO ID
medical treatment for oral biofilm			is_a	treatment	OGMS:0000090
	protect enamel with fluoride		has_part	positive regulation of enamel	GO:0070175
	remove as much plaques as possible		NA	NA	NA
medical treatment for inflammation			is_a	treatment	OGMS:0000090
	nodule formation		equivalent	angiogenesis	GO:0001525
	wound healing		equivalent	wound healing	GO:0042060
medical treatment for pathological bone loss			is_a	treatment	OGMS:0000090
	bone formation		equivalent	bone development	GO:0060348
		decreased RANKL/OPG ratio	has_part	bone remodeling	GO:0046849
		inhibition of osteoclastogenesis	has_part	inhibition of osteoclast differentiation	GO:0045671
		lead apoptosis of preexisting osteoclast	has_part	regulation of macrophage apoptotic	GO:2000109
		maintenance of calcium metabolism in bone	has_part	bone mineralization	GO:0030282
		medical inhibition of osteoclast activity	is_a	inhibition of osteoclast differentiation	GO:0045671
		osteointegration	has_part	bone remodeling	GO:0046849
		osteoimmunology		NA	NA
		prevent binding of RANKL	has_part	RANKL-mediated signaling pathway	GO:0071847
	recovery of bone homeostasis		equivalent	bone remodeling	GO:0046849
		bone resorption		bone resorption	GO:0045453
		crosstalk between osteoblasts and hematopoietic stem cells in bone	has_part	osteoblast development	GO:0002076
		keep balance between bone resorption and bone formation	has_part	regulation of bone remodeling	GO:0046850
		maintenance of calcium metabolism in bone	has_part	bone mineralization	GO:0030282
		maintenance of structural integrity of bone	has_part	bone remodeling	GO:0046849
		osteoimmunology		NA	NA
	clinical treatment				
		regulation of RANKL expression	equivalent	regulation of RANKL production	GO:2000307
	laboratory test for periodontitis		is_a	laboratory test	OGMS:0000056

NA: Not Available
