# Supplementary material for: Development of an Ontology for Periodontitis
Source: J Biomed Semantics. 2015 Jul 1;6:30. doi: 10.1186/s13326-015-0028-y (PMC4488034; doi:10.1186/s13326-015-0028-y)
Supplement: Additional file 3: — This table contains all the mapping results of the classes in processes of medical treatment for periodontitis between PeriO and GO-BP. [file 13326_2015_28_MOESM3_ESM.pdf]

Additional Table 2. Revision from local IDs to OBO IDs of GO, BFO, OGMS, and RO in PeriO.

1) IDs of classes

depth in	before this revision		after this revision	
	label of class	ID	label of class	ID
1	entity	PD:0000001	entity	BFO:0000001
2	continuant	PD:0000002	continuant	BFO:0000002
2	occurrent	PD:0000485	occurrent	BFO:0000003
3	independent continuant	PD:0000229	independent continuant	BFO:0000004
3	specifically dependent continuant	PD:0000003	specifically dependent continuant	BFO:0000020
3	process	PD:0000486	process	BFO:0000015
4	NA	NA	material entity	BFO:0000040
4	NA	NA	realizable entity	BFO:0000017
4	NA	NA	bodily process	OGMS:0000060
4	NA	NA	laboratory test	OGMS:0000056
4	NA	NA	treatment	OGMS:0000090
5	NA	NA	object	BFO:0000030
5	NA	NA	object aggregate	BFO:0000027
5	NA	NA	disposition	BFO:0000016
6	organism part	PD:0000380	extended organism	OGMS:0000087
6	disease	PD:0000141	disease	OGMS:0000031
6	formation of oral biofilm	PD:0000047	multi-species biofilm formation	GO:0044399
6	invasion of bacteria	PD:0001101	entry of bacterium into host cell	GO:0035635
6	pathological bone resorption	PD:0000019	bone resorption	GO:0045453
7	osteoclast differentiation	PD:0000028	osteoclast differentiation	GO:0030316
8	endocytosis	PD:0001085	endocytosis	GO:0006897
8	chemotaxis	PD:0000500	leukocyte chemotaxis involved in inflammatory response	GO:0002232
8	coagulation	PD:0000532	coagulation	GO:0050817
8	phagocytosis	PD:0000537	phagocytosis	GO:0006909
8	multinucleation of osteoclast	PD:0000030	multinuclear osteoclast differentiation	GO:0072674 :

2) IDs of relations

depth in	before this revision		after this revision	
	label of relation	ID	label of relation	ID
1	is:a	NA	is:a	OBO:REL:0000001
1	part:of	PD:0100008	has:part	OBO:REL:0000003
1	has:participant	PD:0100006	has:participant	OBO:REL:0000019
1	subsequent:to	PD:0100009	preceded:by	OBO:REL:0000017
1	located:in	PD:0100007	located:in	OBO:REL:0000008
2	NA	NA	has:agent	OBO:REL:0000021
3	active agent	PD:0100005	has active agent	PD:0100005
3	suppressive agent	PD:0100010	has suppressive agent	PD:0100010

NA: Not available
